# Hospital and patient factors influencing the health status among patients with schizophrenia, thirty days after hospital discharge: multi-level analysis

**DOI:** 10.1186/s12888-020-03001-4

**Published:** 2020-12-14

**Authors:** Anantree Smithnaraseth, Acharaporn Seeherunwong, Rungnapa Panitrat, Mathuros Tipayamongkholgul

**Affiliations:** 1 D.N.S. Candidate, Faculty of Nursing, Mahidol University, Bangkok, Thailand; 2 Department of Mental Health and Psychiatric Nursing, Faculty of Nursing, Mahidol University, 999 Phuttamonthon 4 Road, Salaya, Nakhon Pathom, 73170 Thailand; 3 Faculty of Nursing HRH Princess Chulabhorn College of Medical Science, Chulabhorn Royal Academy, Bangkok, Thailand; 4 Department of Epidemiology, Faculty of Public Health, Mahidol University, Bangkok, Thailand

**Keywords:** Schizophrenia, Health status, Post-discharge, HoNOS, Multi-level logistic regression

## Abstract

**Background:**

The time between discharge from hospital and transition to community and home is a critical period for health status among patients with a mental illness, including patients with schizophrenia. This study aimed to investigate crucial patient factors (patient-level) and hospital factors (hospital-level) affecting health status and see whether patient factor effects on health status vary with hospital factors, 30 days after hospital discharge.

**Methods:**

This is a prospective study of 1255 patients with schizophrenia and their primary caregivers from 13 public mental hospitals across Thailand. Logistic regression and multi-level logistic regression was used to investigate the effects of patient and hospital factors simultaneously on health status, 30 days after hospital discharge.

**Results:**

The intraclass correlation coefficient indicated that 14% of the change in health status was explained by the differences between hospital. Poor health status was identified in 14.26% of patients, 30 days after hospital discharge. The majority of participant patients were male (69.8%), single (71.87%), and the average age was 38.09 (SD = 9.74). The finding also showed that the patient factors; being female (OR_adj_ .53, 95%CI .31,.92), perceived moderate and high levels of positive aspect of caregiving (OR_adj_ .24, 95%CI .14,.42 and OR_adj_ .05, 95%CI .02,.09), perceived readiness for hospital discharge (OR_adj_ .21, 95%CI .13,.33), partial and full adherence to treatment (OR_adj_ .24, 95%CI .14,.42 and OR_adj_ .31, 95%CI .20,.47) showed a reduced likelihood of developing poor health status except substance use (OR_adj_ 1.55, 95%CI .98, 2.44). Hospital factors; discharge planning process and nurse-patient ratio (OR_adj_ 1.64, 95%CI 1.17, 2.30 and OR_adj_ 1.16, 95%CI 1.09, 1.22) showed an increased likelihood of developing poor health status, 30 days after hospital discharge.

**Conclusions:**

Findings provide relevant information on how both patient and hospital factors determine health status. These results might lead to better targeting of mental health service policy and enable more precise information gathering and allocation of resources. However, future research should be more focused and continue investigating the pathways through which hospital factors influence health status post-discharge.

**Supplementary Information:**

The online version contains supplementary material available at 10.1186/s12888-020-03001-4.

## Background

Schizophrenia is a chronic mental disorder that frequently causes problems for the general health of individuals. It affects approximately 1–1.15% of the global population, which afflicts over 20 million individuals worldwide, primarily in young adulthood, and slightly more men than women [1–4]. Schizophrenia is characterized by impairment of insight, judgment, mood, and can produce psychotic symptoms such as hallucinations and delusions, impacting a patient’s global functioning [5–7]. Also, schizophrenia has high social costs due to high treatment costs, loss of productivity, and considerable public assistance [8].

Since the deinstitutionalization policy movement, most patients with a mental illness, including patients with schizophrenia, are now being cared for in the community and at home. In theory, it appeared to be a logical and sound concept to improve the conditions and care of patients with a mental illness. However, when one or more of the individual reasons that supported deinstitutionalization turned out to be false, for either the patient or community, problems began to arise [9]. A key issue affecting patients’ health outcomes is the lack of a smooth transition into integrated and collaborative care planning by the mental health and social care services and inpatient, outpatient, and community mental health care [10]. Research suggests that low transition out of inpatient psychiatric hospitals and inadequate discharge planning may have adverse effects on patients and their families [11]. Moreover, poor transition planning is associated with a higher degree of non-adherence to treatment, worsened prognosis, and increased risk of relapse or readmission [12–17].

When working with patients with a mental illness, mental health care teams, especially nurse staff, must provide direct care in terms of prevention and in promoting and focusing on the patient’s health and potential during admission until discharge and post-discharge. There was a considerable gap in the quality of care offered since the number of professional health workers dealing with mental health in low and middle-income countries (LMICs) is grossly inadequate [18], including in Thailand. Almost half the world’s population lives in countries where, on average, there is only one psychiatrist to serve 200,000 or more people [18]. The World Health Organization in 2015 reported that there were 43.5, 15.2, and 8.7 mental health professionals per 100,000 of the population in samples of the EU, American, and the Western Pacific regions, respectively, and 4.8 per 100,000 of the people in South East Asia. Over 24 staff nurses worked in mental health in the EU, compared to 0.6 in Africa, and 2.6 per 100,000 of the population in South East Asia [19]. Hence, professional mental health staffing, especially nurse staffing, was a critical mental health care system concerned because of its association with quality of care, patient needs, and health outcomes. Even though a growing research interest by nurses, mental health nursing research is still limited, and the nurse to patient ratio effects on health outcomes after hospital discharge are rarely discussed.

Health status in this study refers to all aspects of health (behavioral, physical, clinical, and social) of people who use mental health services due to severe mental illness, including patients with schizophrenia. Monitoring the health status of patients with schizophrenia after hospital discharge may provide health care professionals the information to improve service delivery. Also, monitoring day-after hospital discharge health status for schizophrenia looks to the changes and variations in overall health status. Forward-looking surveillance needs further elucidation but maybe a way to establish or plan better treatment for these patients. The factors associated with health status among patients with schizophrenia after hospital discharge is understood according to the Andersen Healthcare Utilization Model (Andersen’s Emerging Model phase 4, 1995). Andersen’s model focuses on the health service systems where patient needs are met through professional caregiving. It is a multi-level model that incorporates personal and contextual or health service delivery determinants of health service use [20]. Personal or patient factors as predisposing characteristics among patients with schizophrenia that are often associated with health status were young age, male gender, low level of education [5, 21–23], long duration of illness [24–26], and substance use [27, 28]. The positive aspect of caregiving (PAC) from caregivers is an enabling resource that influences treatment adherence and improves health status among patients with schizophrenia [29, 30]. Also, caregivers report lower rates of depression and burden related to daily care activities [31] and improvement in outcome due to long-term adherence to treatment by patients [32]. Finally, the severity of illness at discharge could be measured by readiness for hospital discharge (RHD), as perceived by the patients’ need components. Patients who saw themselves as unready for release showed the highest impairment in health status, including impaired community functioning, more severe psychopathology, more impaired cognitive functioning, and more inadequate psychosocial adjustment [13, 33, 34].

Environment or hospital factors include the health care system and external environment, which refers to the amount and distribution of health service facilities or availability of service delivery that support the individual patient [35]. Previous studies have reported that adequate care provision during hospitalization by staff affects treatment adherence, self-care improvement, and cognitive and social functioning improvements among patients with schizophrenia [11]. Staff to patient ratio was the specific variable that substantially impacted the quality of care concerning the risk of readmission within 30-days and patient’s health outcomes [36–38].

One problem posed by the disorder is the limited number of studies and the lack of investigations into the factors that influence the health status of patients with schizophrenia after hospital discharge. Most of the research has focused on patient factors, and less attention has been given to hospital factors, in particular, the discharge planning process, nurse staffing, and the health status of patients with schizophrenia. Thus, to provide a more comprehensive view and gain insight into the factors that influence health status after hospital discharge, it is necessary to explore how patient and hospital factors contribute to the health status of patients with schizophrenia in thirteen mental hospitals across Thailand. This study aimed to examine the patient factors (patient-level) and hospital factors (hospital-level) influencing health status among patients with schizophrenia 30 days after hospital discharge. Emphasis is placed on exploring the actual effect of these elements on health status, considering the impact of their levels. We hypothesize that patient and hospital factors affect health status among these patients, 30 days after hospital discharge.

## Methods

### Setting

Thailand is a country in South East Asia, with a population of approximately 63 million persons. It has five regions (central, northern, northeastern, eastern, and southern) and seventeen public mental hospitals distributed throughout the country. These hospitals provide 13.8 beds per 100,000 population with thirteen public mental hospitals for adults and four for children and adolescents. All public mental hospitals are organizationally integrated with outpatient facilities [39]. The Mental Health Department (MHD) is a representative of the Ministry of Public Health (MOPH) and is responsible for the implementation and administration of mental health services and issues mental health regulations and notifications for the execution of service delivery. After the health reform in 2012, the role of MHD now includes the development of mental health policy and mental health service regulations at provincial and district levels [40]. This study focuses on 20–59-year-old adult patients with schizophrenia from thirteen public mental hospitals invited to participate in the study. Based on this large population, the appropriate method of participant sampling from different settings used a proportion-to-size sampling method.

### Study participants

The inclusion criteria for the participant patients included: 1) being 20 to 59 years of age; 2) principal or first diagnosis of either schizophrenia (F20.0–F20.9) or schizoaffective disorders (F25.0, F25.1, F25.2, F25.8, F25.9) based on ICD-10; 3) were inpatients of public mental hospitals and whom their psychiatrist had permitted discharge from the hospital to return home during March 2018 to June 2019; 4) living with a close family member or caregiver; 5) able to understand and communicate in Thai, and 6) willing to participate in the study. Cases that were transferred to another inpatient facility due to physical problems were excluded. The primary participant caregivers who had been most involved with participant patients in the last 3 months, living with the patient at home, able to understand and communicate in Thai, were invited to participate in the study as well.

The number of patients with schizophrenia who were discharged from public mental hospitals in Thailand in 2015 was about 37,938 [41]. The Krejcie & Morgan [42] method was used to determine the required sample size. Based on previous evidence, we assumed a 40% dropout rate due to uncompleted questionnaires and the inability to contact patients after hospital discharge. The total required sample size was calculated to be 1500 participating patients and their primary caregivers starting on the day of hospital discharge from thirteen mental hospitals within the period of the study. In the current study, we excluded participants due to questionnaires with missing data (*n* = 54), inconvenience, and inability to contact location phone numbers for the telephone interview 30 days after hospital discharge (*n* = 191), ending up with a sample of 1255 participants and yielding a response rate of 83.66%.

### Measures

Health status was assessed using The Health of the Nation Outcome Scale (HoNOS), developed by the Royal College of Psychiatrists’ Research Unit (CRU) in 1996 and translated into the Thai language by Phuaphanprasert et al. [43]. The HoNOS consists of 12-items, including symptoms, functioning, social relationships, and environmental issues. Each item was rated on a scale of 0 to 4 with 0 meaning no problem, 1 meaning a problem not requiring any intervention, and 2, 3, and 4 corresponded to a “mild,” “moderate,” or “severe” problem, respectively. The total HoNOS is categorized through two cut-off points: 0 = improved clinical (good) health status (0–12) and 1 = worsening (poor) health status (more than 12) [44]. Cronbach’s alpha obtained for the pilot group and total participating patients in the current study was .90. The HoNOS was assessed for participant patients on the day of hospital discharge and 30 days later.

#### Patient factors (patient-level)

The following factors were included in descriptive statistics: gender (male or female), age-group (20–32 yrs., 33–45 yrs. and 46–59 yrs.), educational level (no education, elementary, secondary, vocational, or higher education level), duration of illness (measured in the number of years), substance use (measured dichotomously as no = 0 and yes = 1). These were applied to the participant patients on the day of hospital discharge, except for treatment adherence (not adhering to treatment, partially adhering to treatment, and fully-adhering to treatment), assessed thirty days later.

Readiness for hospital discharge (RHD) of the patients was assessed using the Readiness for Hospital Discharge Scale (RHDS), developed by Wiess et al. [45] and translated into the Thai language by Sriprasong et al. [46]. The RHDS consists of 23-items and four subscales, including personal status, knowledge, coping ability, and expected support. Each item was rated on a scale of 0 to 10 (0 = not ready to 10 = ready to discharge) with higher ratings indicating greater perceived readiness for discharge. Cronbach’s alpha of total scale findings was .93 and .88 from a pilot study and all participants in the current study. The RHDS was administered to participant patients on the day of hospital discharge.

The positive aspects of caregiving by caregivers were assessed using the Positive Aspect of Caregiving (PAC), developed by Tarlow et al. [47]. The PAC consists of 11-items, phrased as statements about the caregiver’s mental and affective state as part of the caregiving experience. Each item was rated on a 5-point ordinal scale, ranging from 1 (strongly disagree) through 5 (strongly agree). We applied the translation processes of forward-translation and then back-translation specified in the WHO guidelines for the PAC. The aggregated values ranged from 11 to 55, divided into three PAC groups (low score 1–25, moderate score 26–40, and high score 41–55). Cronbach’s alpha of the total scale findings was .90–.96 from a pilot study and all the participants in the current study. The PAC was administered to the participating caregivers when the participating patients were discharged from the hospital.

#### Hospital factors (hospital-level)

Data were obtained from the annual report of the MHD and administration nurse reports from thirteen mental hospitals. They included: 1) a hospital profile regarding the number of beds, the number of professional mental health staff (psychiatrists, nurses, psychologists, and social workers) working in each hospital, and the number of patient hospitalizations during the period of the study [48]; 2) discharge planning process is the process of the development of an individualized discharge plan for a patient before leaving the hospital for the home to reduce unplanned readmission to the hospital [49]. Discharge planning can be an individualized intervention or group-based intervention. In this study, the intervention was classified in one of two ways: as an individual and group intervention by a nurse and a group intervention only by mental health care teams. Data were obtained from medical records. The total number of nurses at each unit was collected from administration nurse reports and recorded by the RA working at each setting. Nurse staffing was calculated as the total number of nurses on the day, evening, and night shifts of the unit from each hospital divided by the number of patients who stay in that unit. The average number of nurses and patients was aggregated in the hospital factors as a nurse to patient ratio.

### Data collection

After the approval of the Mahidol University Institutional Review Board, Nursing (COA No.IRB-NS2018/434.0103) and the Mental Health Department Institutional Review Board (DMH-IRB.COA009/2561) for permission to collect the data from thirteen public mental hospitals, under the Ministry of Public Health Administration in Thailand, data collection commenced as 1) The researcher selected the research assistants (RA) who working as nurses in each hospital, then made an appointment to describe the research objectives, research procedures, criteria of samples, instruments and data collection process including human rights protections; 2) the RA attended and observed the researcher collecting data and any misunderstandings about data collection procedures were discussed and reviewed; 3) the RA practiced collecting data at hospitals where they worked while the researcher also observed until they were able to collect data independently.

For participating patients with schizophrenia, the following process was followed: 1) The researcher or RA coordinated the heads or senior nurses of each participating ward to collected data of participating patients on the day of hospital discharge; 2) invited participating patients to a private area, gave them information about the study and the confidentiality of the data, had them sign an informed consent document, and provided them questionnaires to measure patient characteristics, RHD and health status at baseline; 3) after data collection, the second interview date and time was scheduled. Thirty days later, as expected, the participating patients were called to complete the telephone interview to measure their current health status and treatment adherence.

The process for primary caregivers included: 1) The researcher or RA entrusted the heads or senior nurses of each participating ward with the task of collecting data from the participating caregiver in the ward on the day that participating patients were to be discharged; 2) invited participating caregivers to a private area, gave them the information they required about the study, had them sign an informed consent document, and provided a questionnaire to measure PAC. Finally, the researcher or RA gave a small gift to both participating patients and their caregivers for participation. Each questionnaire was given a code number for identification purposes and to assure confidentiality.

### Statistical analysis

Descriptive statistics were used to examine the distribution of participating patients with schizophrenia for demographic and mental hospital characteristics. Logistic regression analysis was used to determine patient and hospital factors on health status and enter the base model of multi-level logistic regression analysis. Multi-level logistic regression analysis is followed with all the significant elements found in the previous univariate analysis to assess their simultaneous effect on the health status. There are three steps: first, we estimated a null model and calculated the intraclass-correlation coefficient (ICC) (Model 1). Secondly, we included patient factors (Model 2) and, finally, had both patient and hospital factors in addition to hospital-specific random effects (Model 3). At each step, Akaike’s Information Criterion (AIC) was calculated, and the model with the lowest AIC value chosen as the final model that Hosmer & Lemeshow showed as an acceptable model fit. The level of significance of the results was *p*-value< 0.05. All analyses used the program STATA/IC version 16.1.

## Results

### Descriptions of the demographic characteristics related to health status

Table 1 details the demographic characteristics of a total of *N* = 1255 patients with schizophrenia relative to their health status, at baseline and 30 days after discharge from thirteen mental health hospitals. Of all patients, 5.02 and 14.26% had poor health status at baseline and 30 days after release, respectively. The health status at baseline showed an average HoNOS score of 3.81 and 6.47 at 30 days after discharge among participant patients. On average, the total HoNOS increased by 2.66 from the baseline, indicating worsening or poor health status. Also, the health status varied with demographic characteristics; for example, males were more likely to have poor health status than females. Having poor health status was more frequently reported in the younger age groups, single status, uneducated/ elementary and secondary/vocational, and unemployed, than among older, married status, and those having a higher educational level and employment. Poor health status was also more prevalent among those who frequently or ever used drugs or alcohol. Even those who claimed they were ready for hospital discharge acknowledged that they had poor health status after hospital discharge. Moreover, it was confirmed by the findings that the participant patients who did not adhere to treatment, although graded mild or moderate of PAC from their caregivers, were likely to poor health status after hospital discharge.

**Table 1:**
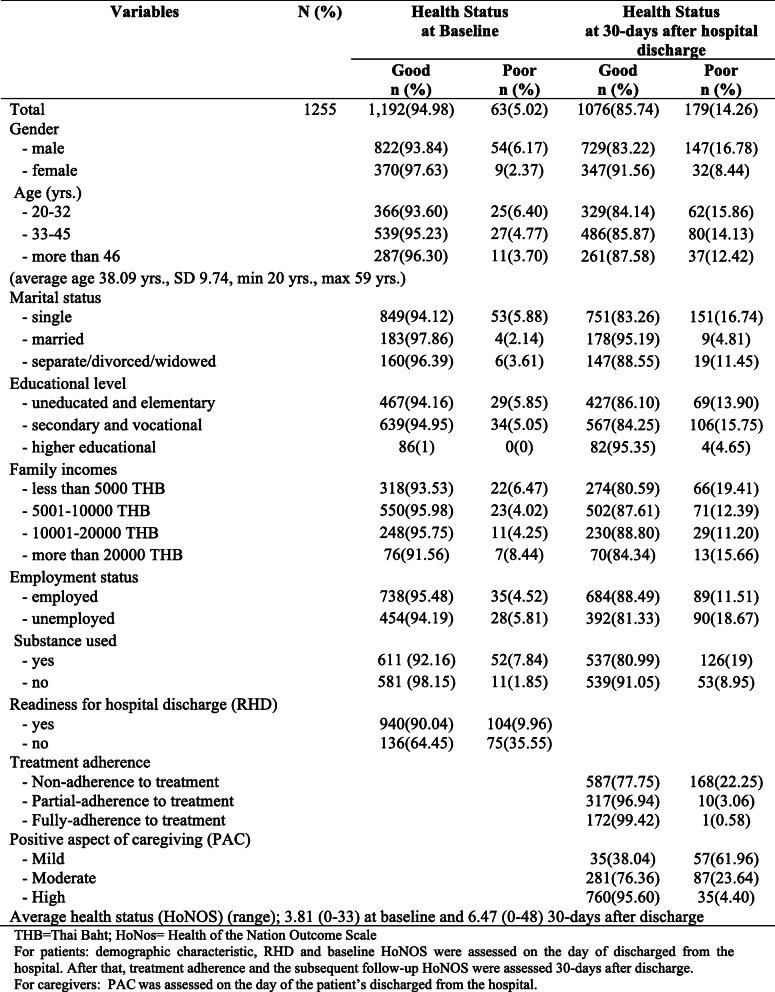
Demographic characteristics of patient with schizophrenia based on health status, at baseline and thirty days after hospital discharge

Information regarding the thirteen mental hospital characteristics is shown in Table 2, as presented in the Supplementary Material. The number of beds varied from area to area in Thailand, with the northeast region having smaller hospitals (90 beds) while the central region had larger hospitals (500–750 beds). More than half of the mental hospitals (*n* = 8) provided discharge planning processes that focused on either individual discharge or group discharge by nurses, and administration of service delivery as acute care units (acutely ill patient care until hospital discharge) (61.53%). The central region had the most professional mental health staff (PMHS) (33.21%), followed by the northeast (30.29%), while the eastern region had fewer PMHS than any other part of Thailand (2.83%). The average nurse-patient ratio on the day shift was 8.41 (SD 2.34).

**Table 2:**
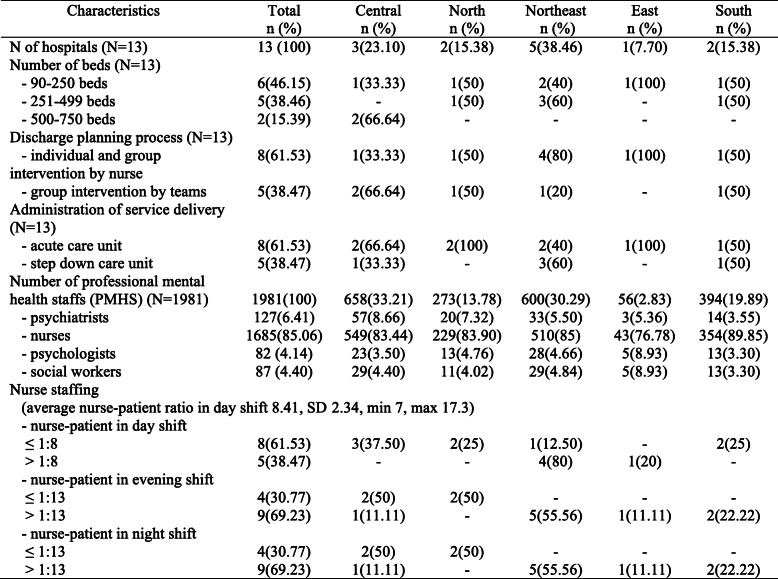
Mental hospital characteristics

### Patient and hospital factors influencing health status among patients with schizophrenia, thirty days after hospital discharge

The patient factors influencing health status when the participating patients were female (OR_adj_ .53, 95%CI .31,.92), moderate and high level of PAC from primary caregivers (OR_adj_ .24, 95%CI .14,.42 and OR_adj_ .05, 95%CI .02,.09), perceived RHD (OR_adj_ .21, 95%CI .13,.33), partial and full adherence to treatment (OR_adj_ .24, 95%CI .14,.42 and OR_adj_ .31, 95%CI .20,.47) showed reduced opportunity of developing poor health status at statistical significance except for substance use (OR_adj_ 1.55, 95%CI .98, 2.44). For hospital factors, the discharge planning process based upon group intervention by teams and the nurse-patient ratio showed an increased opportunity for developing poor health status at statistical significance (OR_adj_ 1.64, 95%CI 1.17, 2.30, and OR_adj_ 1.16, 95%CI 1.09, 1.22) (Table 3).

**Table 3:**
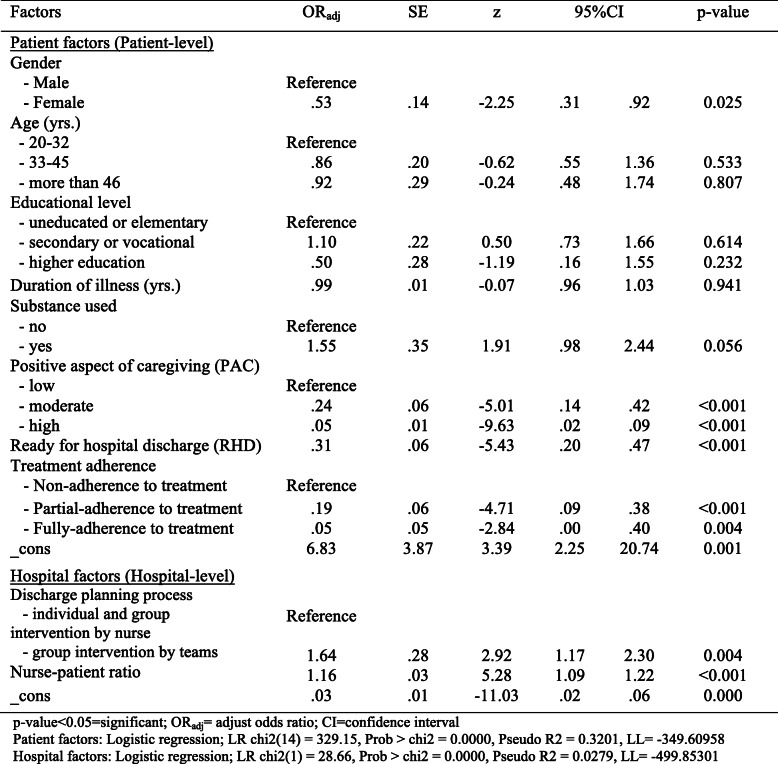
Logistic regression analysis of patient and hospital factors influencing health status, thirty days after hospital discharge

**Fig. 1:**
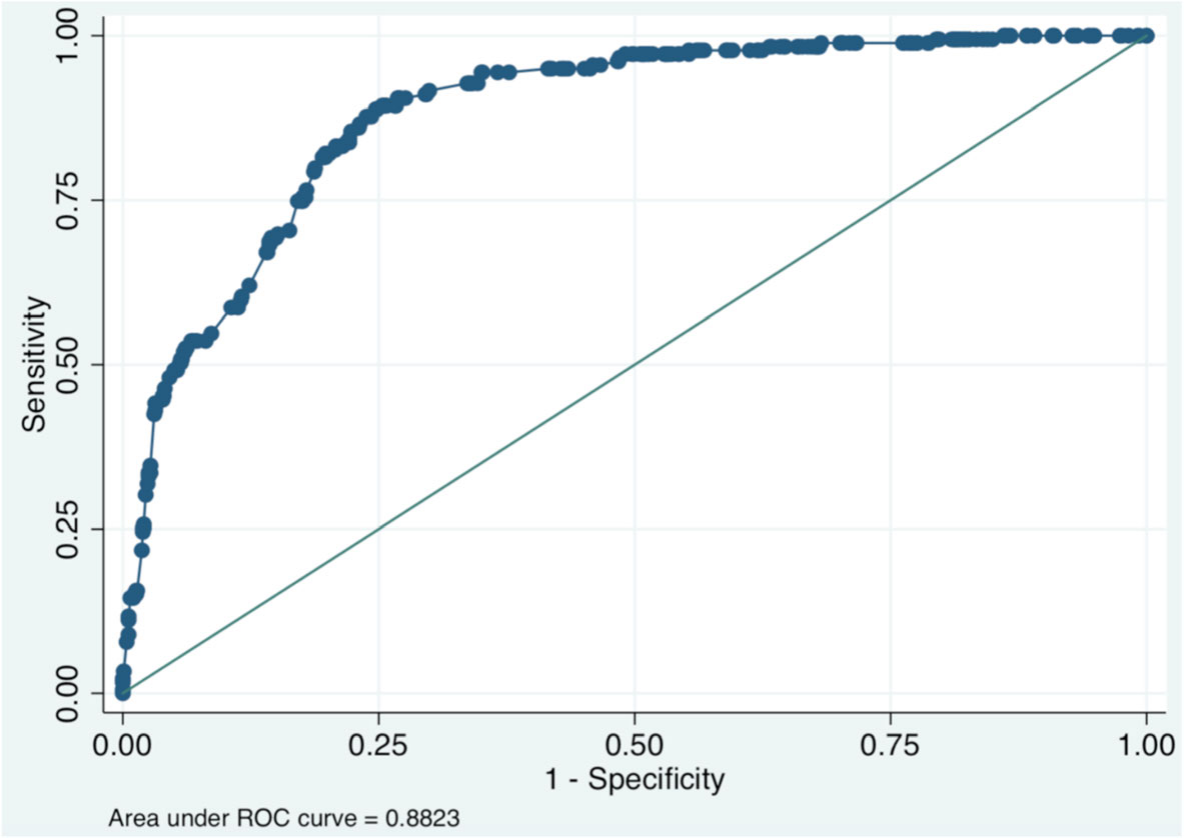
The ROC curve for predictive variables associated with the health status among patients with schizophrenia, thirty days after hospital discharge

The ROC curve for the predictive variables associated with the health status among the participating patients is presented in Fig. 1. Under the null hypothesis (straight diagonal line), the area under the curve is 0.5; the two factors improved the area under the curve to 0.8823. This improvement indicated that the model provides better predictive accuracy than obtained by chance.

### Multi-level model influencing the health status among patients with schizophrenia, thirty days after hospital discharge

The final results of the multi-level model are presented in Table 4. The method used by Austin & Merlo was based upon a three-step multi-level logistic regression model [50]. The ICC calculated from Model 1 is 0.14 (*p* < 0.001), which indicates that 14% of the change in health status is explained by the differences between mental hospitals or settings. The remaining 86% of the variance resided within hospitals. Model 2 includes patient factors that reveal a statistically significant correlation with health status in the logistic regression model (gender, substance use, PAC, RHD, and treatment adherence). The regression coefficients for all of the patient factors are all significant except for substance use. In the final model, Model 3, hospital factors were included and selected after validating other models because this simple model presented the quality of the fixed-effect model with the lowest values of AIC (Akaike information criteria) and Log-likelihood (LL) than as compared to other models. The results of the best model (Model 3) show that patient levels do have an effect on the health status and do vary by hospital level. Female participant patients decrease the odds of poor health status by .75 points (*p* < 0.05), using males as the reference. Also, when the score of PAC from primary caregiver increased by one unit, the poor health status among patients with schizophrenia was reduced by 1.33 and 3.11 points (*p* < 0.001). When the participant patients were ready for hospital discharge, poor health status was reduced by 1.09 points (*p* < 0.001). The poor health status among participant patients with partial or full adherence to treatment was decreased by 1.67 (*p* < 0.001) and 2.84 (*p* < 0.05). Moreover, poor health status among participating patients increased by 0.11 points when the average ratio of nurse to patient increased (*p* < 0.05).

**Table 4:**
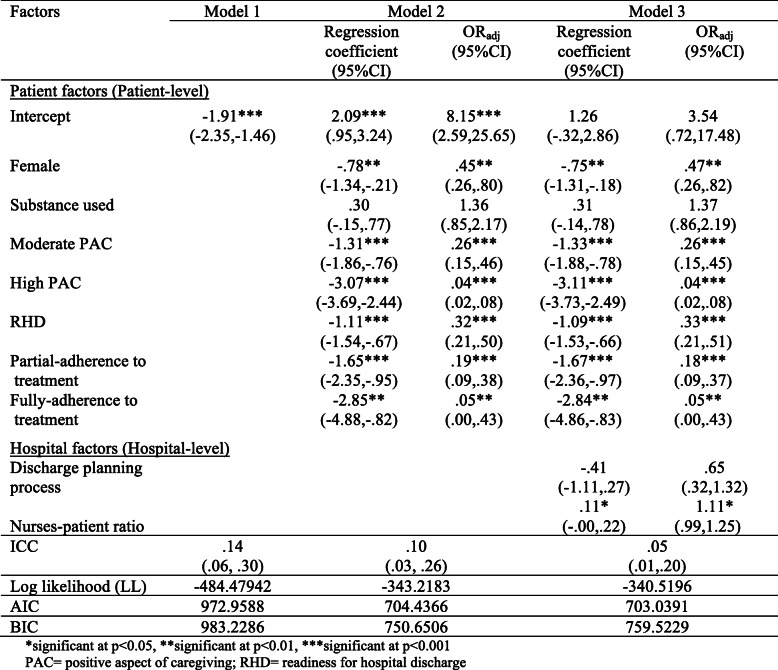
Estimated regression coefficients, odd ratio and variance components for the multilevel logistic regression models.

## Discussion

This study investigates the patient and hospital factors influencing health status among patients with schizophrenia, 30 days after hospital discharge. Of the 1255 patients with schizophrenia, 14.26% had worsening or poor health status, 30 days after hospital discharge. These findings go hand in hand with results in previous studies in other countries [51–54]. Nevertheless, the reasons for the higher chance of developing poor health status are not clear, but, generally, differences in prevalence are related to study factors, periods of measurement, operational definitions, and measurement use, as well as the targeted populations.

Our findings revealed five patient factors (gender-difference, substance use, PAC from primary caregivers, perceived RHD, and treatment adherence) significantly influenced the health status among patients with schizophrenia, 30 days after hospital discharge. For gender-difference, the female gender was a significant predictor of health status. This finding is consistent with previous studies in which women with schizophrenia achieved better health status or health conditions than men [2, 5, 24, 55–57]. Therefore, the mental health service system should be sensitive to differences in gender to meet patients’ specific needs and potentially improve outcomes. Moreover, substance use was also a significant predictor of health status. Previous studies have reported that past use of psychoactive drugs by patients with mental illness are a factor in treatment adherence and eventual health status. It is associated with the deterioration of health status, the risk for future non-adherence to treatment, relapse, and re-hospitalization [27, 28], and this study supports those findings. These findings might result from the mechanism or interactions between substance or alcohol use and intake of psychotropic medications on pharmacokinetics or pharmacodynamics, which can lead to adverse consequences. PAC from primary caregivers was also a significant predictor that influenced health status among participant patients. When caregivers had positive views and demonstrated positive aspects of caregiving, they had lower rates of depression and reduced upset related to care behaviors of patients. This behavior resulted in a lighter caregiver workload of daily care activities, improved outcomes from long-term adherence to treatment, and an overall improvement in the health status of patients [31, 32, 58]. Thus, the benefits of caregiver positive support underline the importance of the family system and community as a source of bonding, belonging, and aid in this environment [59]. The study also found that the participating patients who claimed they were ready for hospital discharge manifested similar health status effects as in previous studies [60–62]. However, the association between the readiness for hospital discharge and health status post-discharge remains unclear in individuals with schizophrenia. This lack of clarity is because most clinical trial literature focuses on symptoms, functional measures, and judgment about possible discharge made by health care providers. As expected, our findings showed non-adherence to treatment was influencing the health status among patients with schizophrenia in accord with past literature [63–67]. This finding sheds light on participants that adhere to treatment and present good health status after hospital discharge.

The findings of this study also identified two hospital factors (the discharge planning process and nurse staffing-patient ratio) that significantly influenced the health status among patients with schizophrenia after discharge from the hospital to their home. Since psychiatric nurses have close relations with patients throughout the treatment plan and the discharge planning process, they can play a crucial role in dealing with patients to improve treatment adherence, health status, and post-discharge outcome. However, there has been an inequitable distribution of PMHS in Thailand, which exists in several other countries. This maldistribution inevitably affects patients’ health outcomes [68–71]. Our study findings amplify the findings of previous studies by showing the effectiveness of adequate nurse staffing, not only regarding care on the unit but after hospital discharge. A possible lesson from these findings is that it is vital to have an adequate ratio of providers to patients in psychiatric units, enabling nurses to devote more time to therapeutic interaction with the patients. They can look forward to applying more effective activity therapy such as psychoeducation or psychotherapy, for improving relationships with the patients. Patients with a mental illness, including patients with schizophrenia, having favorable views of and useful insights into their ailment during admission, has been shown to encourage better treatment adherence and punctual attendance at appointments. This attitude contributes to an improved health status after hospital discharge and a low risk of readmission [13, 27, 59]. Although there is a growing interest in mental health nursing research, there is still a limited evidence base. There is a lack of information available to determine the right number of staff nurses or the appropriate ratio required to ensure good quality of care in the inpatient psychiatric unit and ensure patient outcomes. Limited empirical evidence is available to determine the right mix of providers and the best approach to treating patients in the inpatient psychiatric unit.

This study concludes that both patient and hospital factors have a statistically significant influence on the health status among patients with schizophrenia after hospital discharge. Previous studies also indicated that health outcomes could be predicted from both patient factors and a combination of contextual or hospital factors, which could vary considerably [72–74]. However, results may differ by other hospital variables, factors such as unit type (acute care unit or rehabilitation unit), the type of mental health professionals, and by skill mix team. Further research needs to take into account these hospital variables.

### Strengths and limitation

This study provides the first multi-level findings and an evidence base for the health status among patients with schizophrenia, 30 days after hospital discharge in Thailand. The results indicate the best predictive model for health status when combining factors. Many existing multi-level studies in Thailand have shown a greater focus on individual and optimal interventions provided for the patients. However, both patient and hospital factors are crucial in determining health status. These considerations have important implications for mental health policymakers and enable us to obtain clarity through precise information and better allocation of resources.

There are several limitations to this study. The first limitation is that some of the data was derived from self-reports or subjective, produced by participating patients and caregivers themselves. There is a personal element present here that may lead to bias and an under or over-estimation of the health status among the participants. The second limitation is that this study could not identify the exact working nurse-patient ratios because the number of head nurses, nurse supervisors, and deputy-head nurses in the psychiatric unit was included. Finally, providing only the number of nurses may not be enough, and effects may remain unclear about the association between the staff nurse and outcomes due to unit type, staff working experience, type of mental health professionals involved, and skill mix. Thus, future studies are required to improve the overall elements of the findings and establish what might be the optimal level of staffing.

## Conclusion

Our findings provide evidence that some patient and hospital factors influence health status among patients with schizophrenia, 30 days after hospital discharge. This finding indicates the importance of enabling resources to primary caregivers for positive caregiving, continuing care with treatment, an appropriate discharge planning process, and adequate nurse staffing-patient ratio as effective strategies for improving patient health status and post-discharge outcomes.

## Supplementary Information

**Additional file 1.**

## Data Availability

The datasets used and analyzed during the current study are available from the School of Graduate Study, Mahidol University, on reasonable request.

## Abbreviations

PAC: Positive aspect of caregiving
RHD: Readiness for hospital discharge
PMHS: Professional mental health staffs
MHD: Mental health department
