# Supplementary material for: Hospital and patient factors influencing the health status among patients with schizophrenia, thirty days after hospital discharge: multi-level analysis
Source: BMC Psychiatry. 2020 Dec 14;20:592. doi: 10.1186/s12888-020-03001-4 (PMC7737347; doi:10.1186/s12888-020-03001-4)
Supplement: Supplementary file 1 — Additional file 1. [file 12888_2020_3001_MOESM1_ESM.pdf]

Hospital and patient factors influencing the health status among patients with schizophrenia, thirty days after hospital discharge: Multilevel analysis

Table 2 Mental hospital characteristics

Characteristics	Total n (%)	Central n (%)	North n (%)	Northeast n (%)	East n (%)	South n (%)
N of hospitals (N=13)	13 (100)	3(23.10)	2(15.38)	5(38.46)	1(7.70)	2(15.38)
Number of beds (N=13)						
90-250 beds	6(46.15)	1(33.33)	1(50)	2(40)	1(100)	1(50)
251-499 beds	5(38.46)	-	1(50)	3(60)	-	1(50)
500-750 beds	2(15.39)	2(66.64)	-	-	-	-
Discharge planning process (N=13)						
individual and group intervention by nurse	8(61.53)	1(33.33)	1(50)	4(80)	1(100)	1(50)
group intervention by teams	5(38.47)	2(66.64)	1(50)	1(20)	-	1(50)
Administration of service delivery (N=13)						
acute care unit	8(61.53)	2(66.64)	2(100)	2(40)	1(100)	1(50)
step down care unit	5(38.47)	1(33.33)	-	3(60)	-	1(50)
Number of professional mental health staffs (PMHS) (N=1981)	1981(100)	658(33.21)	273(13.78)	600(30.29)	56(2.83)	394(19.89)
psychiatrists	127(6.41)	57(8.66)	20(7.32)	33(5.50)	3(5.36)	14(3.55)
nurses	1685(85.06)	549(83.44)	229(83.90)	510(85)	43(76.78)	354(89.85)
psychologists	82 (4.14)	23(3.50)	13(4.76)	28(4.66)	5(8.93)	13(3.30)
social workers	87 (4.40)	29(4.40)	11(4.02)	29(4.84)	5(8.93)	13(3.30)
Nurse staffing (average nurse-patient ratio in day shift 8.41, SD 2.34, min 7, max 17.3)						
- nurse-patient in day shift						
≤ 1:8	8(61.53)	3(37.50)	2(25)	1(12.50)	-	2(25)
> 1:8	5(38.47)	-	-	4(80)	1(20)	-
- nurse-patient in evening shift						
≤ 1:13	4(30.77)	2(50)	2(50)	-	-	-
> 1:13	9(69.23)	1(11.11)	-	5(55.56)	1(11.11)	2(22.22)
- nurse-patient in night shift						
≤ 1:13	4(30.77)	2(50)	2(50)	-	-	-
> 1:13	9(69.23)	1(11.11)	-	5(55.56)	1(11.11)	2(22.22)
